# Recent advances in the treatment and prevention of peripheral neuropathy after multiple myeloma treatment

**DOI:** 10.1002/ibra.12132

**Published:** 2023-09-06

**Authors:** Dan Wen, Song Cao, Yonghuai Feng

**Affiliations:** ^1^ Department of Hematology Affiliated Hospital of Zunyi Medical University Zunyi Guizhou China; ^2^ Department of Anesthesiology Affiliated Hospital of Zunyi Medical University Zunyi China; ^3^ Department of Pain Medicine Affiliated Hospital of Zunyi Medical University Zunyi China

**Keywords:** chemotherapy, mechanism, multiple myeloma, peripheral neuropathy, treatment

## Abstract

The incidence of multiple myeloma (MM) is increasing year by year, requiring chemotherapy drugs to control the condition. With the advent of new proteasome inhibitors, immunomodulators, and monoclonal antibodies, the prognosis of patients has improved significantly. However, peripheral neuropathy caused by drugs limits the dose and duration of treatment, which seriously affects patients' quality of life and treatment outcome. Although the neuropathies induced by chemotherapy drugs have attracted much attention, their mechanism and effective prevention and treatment measures are not clear. Therefore, how to alleviate peripheral neuropathy caused by drugs for treatment of MM is a key issue in improving patients' quality of life and prolonging their survival time, which have some clinical value. In this paper, we review the current research on the pathogenesis, pharmacological and nonpharmacological treatment, and prevention, which expects to present instruction for peripheral neuropathy after treatment of MM.

## Graphical abstract

We focus on the mechanism and treatment of multiple myeloma chemotherapeutic peripheral neuropathy. Current research suggests that bortezomib‐induced peripheral neuropathy (BIPN) is associated with neuroinflammation, endoplasmic reticulum and mitochondrial damage, and apoptosis. Thalidomide‐induced peripheral neuropathy (TIPN) is mainly associated with antiangiogenesis, axonal degeneration, and neuroinflammation. However, the exact mechanism is not yet clear. Currently, in clinical practice, we need to focus on the treatment of their symptoms, mainly nerve‐nourishing drugs and pain medication. Commonly prescribed analgesics are not effective in neuropathic pain. Several studies have found that drugs such as duloxetine, docosahexaenoic acid and alpha‐lipoic acid, methylcobalamin, glutathione, dexanabinol, anticonvulsants, antidepressants, cannabinoids, and paeoniflorin, as well as nonpharmacological treatments such as exercise, acupuncture, nerve blocks, and electrical stimulation, are effective in the prevention and treatment of CIPN, but there are no highly recommended therapies for the prevention or treatment of CIPN.

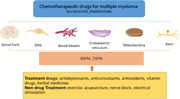

## 1 INTRODUCTION

Multiple myeloma (MM) is a malignant plasma cell hematological disease in the bone marrow, which accounts for 1% of all tumors and 10% of hematological malignancies.^1^ It is the second most common hematologic malignancy. However, MM remains a largely incurable plasma disease. Five major categories of drugs used in the treatment of multiple myeloma, including proteasome inhibitors, immunomodulators, monoclonal antibodies, nuclear output inhibitors, and steroid hormones, have led to very significant effects but at the same time brought some adverse events such as peripheral nerve injury, which should be emphasized.^2^

Chemotherapy‐induced peripheral neuropathy (CIPN) is a progressive, persistent, and often irreversible adverse reaction of many antineoplastic drugs. The main classes of chemotherapeutic agents associated with peripheral neuropathy (PN) are platinum drugs, paclitaxel, periwinkle alkaloids, proteasome inhibitors, and immunomodulators, while those associated with MM are mainly proteasome inhibitors and immunomodulators. CIPN can progress from acute to chronic and may even worsen or only partially relieve after stopping treatment.^3^, ^4^, ^5^ Sensory toxicity is the main feature because the dorsal root ganglion (DRG) contains the cell body of sensory neuron, and the DRG has a porous capsule, which is easier to penetrate than the spinal cord.^6^ CIPN severely affects the quality of life and chemotherapy response rate of MM patients.^7^ Although chemotherapy drug‐induced neuropathy is a matter of great concern, the mechanism of its occurrence and effective measures to prevent and treat are still not very clear.

In this review, we will describe the pathogenesis of neuropathy caused by proteasome inhibitors and immunomodulators, which are two major therapeutic drugs for MM. In addition, we will also illuminate the current prevention and treatment strategies for peripheral neuropathy, which may improve the quality of life and indirectly prolong patients' overall survival rate, and provide an outlook on the future direction of neuropathy treatment after MM.

## 2 THE PATHOGENESIS OF CIPN

### 2.1 Proteasome inhibitor‐induced CIPN

In the current treatment regimens, the proteasome inhibitor bortezomib is the most common chemotherapeutic agent causing PN in MM treatment.^8^ Bortezomib, the first generation of proteasome inhibitor, is commonly used in the chemotherapy of MM and mantle cell lymphoma.^9^ Bortezomib‐induced peripheral neuropathy (BIPN) is an axonal sensorimotor neuropathy characterized by sensory loss, including neuropathic pain, and motor weakness in the distal upper and lower extremities.^10^ In a phase III clinical trial, the median incidence of sensory CIPN in patients given bortezomib was 37.8% and the median incidence of severe neuropathy (grade 3–4) was 8%, with a high incidence of BIPN associated with increased cumulative dosing levels, intravenous administration, and thalidomide coadministration.^11^

**Figure 1:**
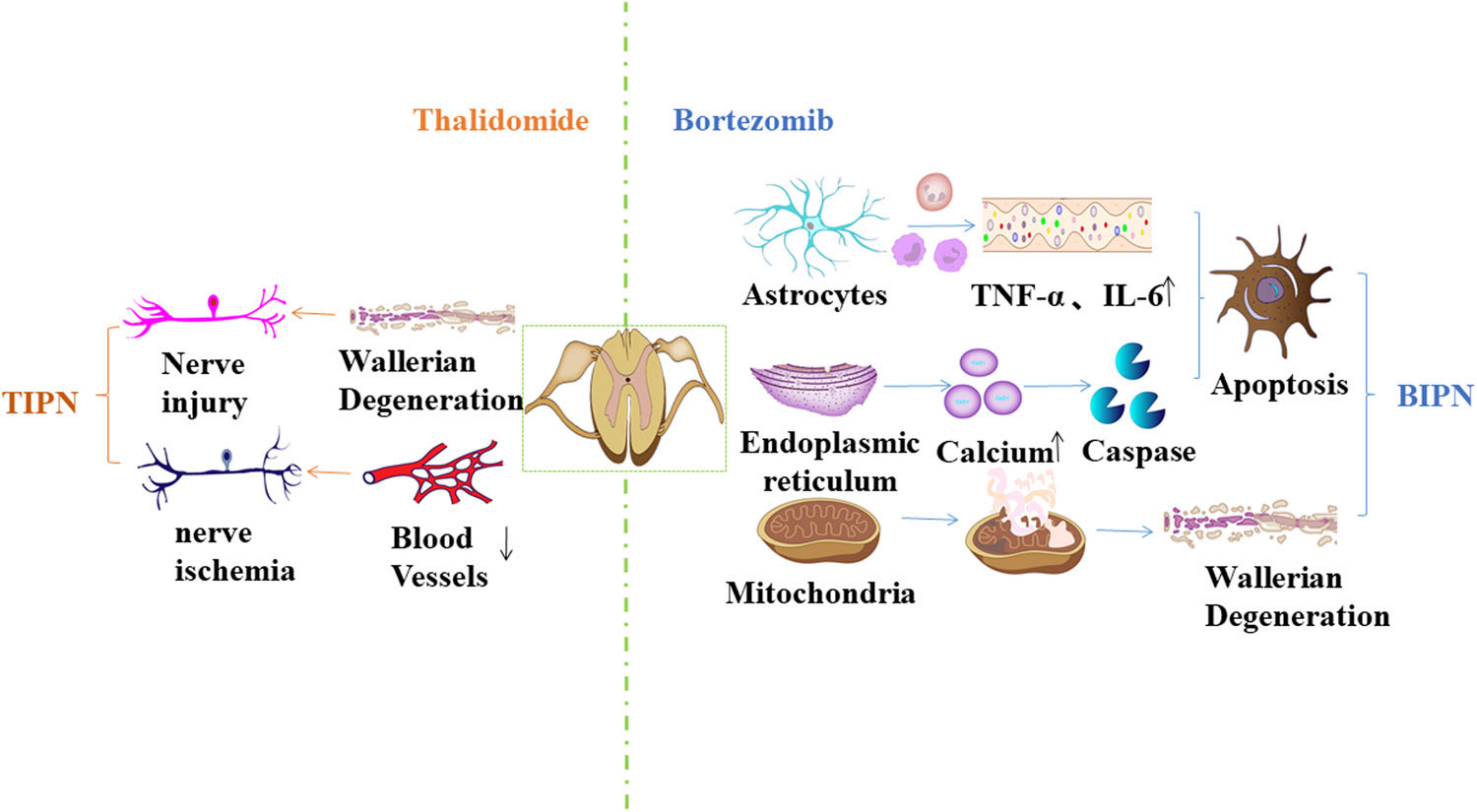
The current hypothesis for the pathogenesis of BIPN and TIPN. Bortezomib leads to glial cell activation, increased production of inflammatory factors, and release of calcium ions from the endoplasmic reticulum to activate apoptotic proteins, ultimately leading to neuronal apoptosis, as well as mitochondrial damage, leading to axonal degeneration. Thalidomide is able to induce degeneration of neuronal cells and exert direct toxic effects on DRG neurons. Further, thalidomide causes capillary damage in nerve fibers while resisting angiogenesis, an effect that reduces capillary production and leads to secondary nerve fiber ischemia and hypoxia. BIPN, bortezomib‐induced peripheral neuropathy; TIPN, thalidomide‐induced peripheral neuropathy. [Color figure can be viewed at wileyonlinelibrary.com]

The main molecular target of bortezomib is 26S proteasome, an adenosine triphosphate‐dependent multidomain proteolytic complex, which is widely expressed in eukaryotic cells and is responsible for the degradation of most short‐lived and long‐lived proteins. It plays a role by specifically and reversibly binding proteasome 26S subunits to inhibit the proteasome ubiquitin pathway, leading to cell cycle inhibition and apoptosis.^12^, ^13^ Due to the blood–brain barrier, bortezomib does not penetrate the central nervous system (CNS). However, it accumulates in DRG neurons and indirectly causes CNS dysfunction, including glial cell activation, mitochondrial and endothelial network damage, and inflammation.^9^, ^14^, ^15^ A study in mice showed that the effects of bortezomib lead to oxidative stress, mitochondrial dysfunction, endoplasmic reticulum stress, and apoptosis.^16^ Bortezomib induces aerobic glycolysis in sensory neurons, which may lead to metabolite efflux, sensitizing primary sensory afferents, and ultimately causing neuropathic pain.^17^ In addition, tubulin acetylation was observed in cell lines, and these alterations in intracellular tubulin kinetics may lead to impaired axonal transport.^12^ Neuroinflammation is one of the main mechanisms of CIPN, and bortezomib increases pro‐inflammatory cytokines (e.g., TNF‐α and IL‐6) and downregulates anti‐inflammatory cytokine IL‐10 in the DRG and spinal cord^18^ (Figure 1). Reduced nerve growth factor and brain‐derived growth factor are responsible for altered neuronal cell survival; however, it may be related to NF‐κB pathway inhibition.^19^ In addition, abnormal signaling pathways and single‐nucleotide polymorphism (SNP) changes in genes may be associated with the occurrence of BIPN. Activation of NOX2‐driven reactive oxygen species‐mediated mTORC1 pathway by bortezomib leads to apoptosis of primary DRG neurons.^20^ It can also cause an increased spinal cord protein kinase C (PKC) phosphorylation and a presynaptic membrane translocation, which leads to glutamate release and bortezomib‐induced sensory hypersensitivity.^21^, ^22^ BTZ causes axonal degeneration through dual mechanisms; the main mechanism is aseptic α and Toll/interleukin‐1 receptor motif 1 (SARM1), while the second mechanism is related to caspase‐3‐mediated apoptosis pathway, leading to axonal degeneration in cultured DRG neurons.^23^ It was found that BIPN mainly involves the dysfunction of nerve fibers, which is the cause of abnormal pain signal transmission, and the main dysfunctional nerve fibers are small myelinated fibers (Aδ fibers), unmyelinated fibers (C fibers), and large myelinated fibers (Aβ fibers).^24^ Previous studies have shown that bortezomib may cause cytoplasmic vacuolation of Schwann cells, and the damaged Schwann cells induce peripheral nerve demyelination and neuropathic pain.^25^ Although there are many studies on the mechanism of BIPN, the exact mechanism is not well understood.

### 2.2 Immunomodulators induced CIPN

Thalidomide, a synthetic glutamate derivative, was marketed in 1956 as a sedative antiemetic for severe pregnancy‐related emesis and was withdrawn from the market in 1961 due to severe teratogenicity. However, due to its potent anticancer effects, especially in MM, thalidomide has been found to be important.^26^ Symptoms of thalidomide‐induced peripheral neuropathy (TIPN) include symmetrical numbness, tingling, burning, and sensitivity to touch and heat, accompanied by sensory sensitivity and sensory abnormality, which is manifested by the glove‐ and stocking‐like distribution. The TIPN incidence rate varies widely, ranging from 11% to 75%, depending on the dose and the duration of the exposure.^3^ Other studies have reported an overall incidence of TIPN of 10% in patients with myeloma, and of these patients, approximately 95% will resolve on their own.^27^ For oral administration of thalidomide, the dose range is 100–1000 mg per day. It is mainly spontaneously hydrolyzed, so there is no need to adjust the dosage in the event of liver or kidney dysfunction.^26^ The combination of bortezomib and thalidomide results in higher rates of neuropathy and is more pronounced, reversible, length dependent, sensory rather than motor, and mainly polyneuropathy of axons and involves large fibers rather than small fibers.^28^ Owing to the high risk of peripheral neuropathy in patients treated with thalidomide, a routine electrophysiological study in all patients with MM is essential to diagnose neuropathy at an early stage.

Thalidomide induces neuronal cell degeneration and has a direct toxic effect on DRG neurons. Drugs used to treat MM can cause axonal lesions in neuronal cells and damage DRG neuronal cell bodies. Therapeutic drugs are more likely to damage neuronal cells with long axons, but CNS is rarely damaged when treated with anti‐MM drugs. Thalidomide affects the axons of sensory and motor nerves, causing neurotoxicity and damage to nerve fibers. Capillary injury in nerve fibers is accompanied by antiangiogenesis, an effect that reduces capillary production and leads to secondary nerve fiber ischemia and hypoxia (Figure 1).^29^ In addition, thalidomide downregulated TNF‐α and inhibited nuclear factor κβ, causing dysregulation of neurotrophic factor activity and thus leading to neuronal demyelination and accelerated neuronal cell death.^30^

## 3 CLINICAL MANIFESTATIONS

Patients with CIPN display a range of symptoms, including sensory abnormalities, neuropathic pain, muscle weakness, and diminished reflexes. Sensory abnormalities may manifest as tingling, prickling sensations, and numbness. Additionally, severe cases may involve cold extremities, muscle spasms, and movement difficulties. CIPN is characterized by symmetric pain and numbness in a “glove–sock” distribution across all four limbs. Fine motor abilities, such as buttoning clothes or tying shoelaces, can be affected. In rare instances, patients may experience muscle weakness, abdominal pain, diarrhea, and constipation. The duration of CIPN symptoms can extend from several months to years after treatment discontinuation. Additionally, physical examination findings play a crucial role in CIPN diagnosis. Neurological signs include decreased or absent sensation, muscle weakness, muscle atrophy, diminished reflexes, and pathological reflexes. Treatment interventions can be tailored based on specific symptoms and physical examination findings to address sensory abnormalities, manage pain, and promote motor rehabilitation. These interventions aim to alleviate symptoms and improve the patient's quality of life.

## 4 PREVENTION AND TREATMENT

**Figure 2:**
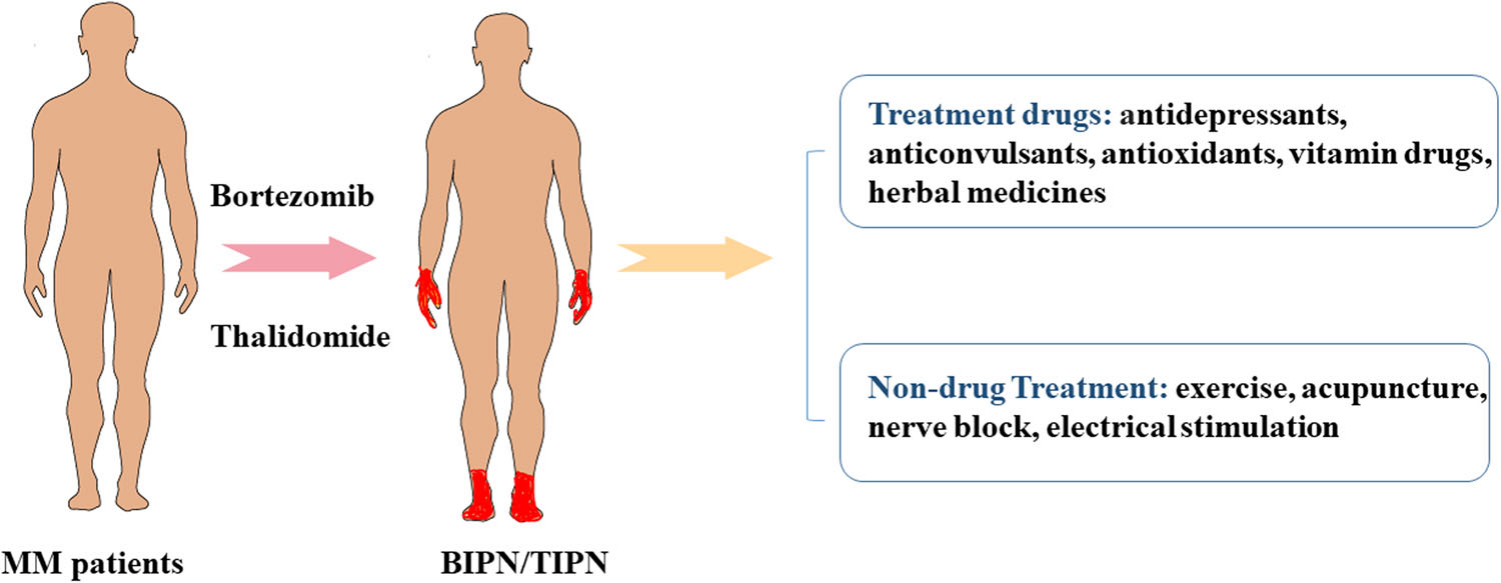
Treatment methods for CIPN in MM patients. Pharmacological treatments are mainly nutritional nerve drugs and analgesic drugs, including duloxetine, docosahexaenoic acid and alpha‐lipoic acid, methylcobalamin, glutathione, dexanabinol, anticonvulsants, antidepressants, cannabinoids, and paeoniflorin, as well as nonpharmacological treatments such as exercise, acupuncture, nerve blocks, and electrical stimulation. BIPN, bortezomibinduced peripheral neuropathy; MM, multiple myeloma; TIPN, thalidomide‐induced peripheral neuropathy. [Color figure can be viewed at wileyonlinelibrary.com]

As the pathogenesis of MM chemotherapy‐induced peripheral neuropathy has not been elucidated, no highly recommended methods of prevention and treatment have been reported to date. Current clinical practice focuses on the treatment of their symptoms, mainly nerve‐nourishing drugs and analgesics, with commonly used analgesics not working for neuropathic pain. Reducing the dose of chemotherapeutic agents or the cycles of treatment may partially result in the improvement of neurological symptoms, but it also inevitably ameliorates the prognosis of MM. Several studies have found that drugs such as duloxetine, docosahexaenoic acid and alpha‐lipoic acid, methylcobalamin, glutathione, dexanabinol, anticonvulsants, antidepressants, cannabinoids, and paeoniflorin (Table 1), as well as nonpharmacological treatments such as exercise, acupuncture, nerve blocks, and electrical stimulation, are effective in the prevention and treatment of CIPN, but there are no highly recommended therapies for the prevention or treatment of CIPN (Figure 2).^31^ Various opioids (including oxycodone, morphine, fentanyl, and tramadol) are also used clinically for other symptomatic interventions. Nonsteroidal antiinflammatory drugs (NSAIDs), including celecoxib, rofecoxib, ibuprofen, and acetaminophen, and vitamins and nutritional supplements, such as vitamin B6 and vitamin C, are also used to relieve neuropathic pain in BINP.^19^ However, the therapeutic effects of these drugs do not meet the clinical needs. Therefore, it is necessary to find some new therapies to prevent and manage CIPN symptoms.

**Table 1:** Classification of drug therapy for CIPN.

Categories of drugs	Drug names	Therapeutic characteristics	Advantages	Disadvantages	References
Antidepressants	Duloxetine amitriptyline	Reducing pain, anxiety, and depression in CIPN patients	Safe and long‐lasting efficacy	Side effects and individual differences of antidepressants and interactions with other drugs	[31, 32, 33]
Anticonvulsants	Gabapentin pregabalin	Reduced neural excitability	Well tolerated, safe, and sustained efficacy	Side effects and individual differences of antidepressants and interactions with other drugs	[8, 34, 35, 36]
Antioxidants	Glutathione	Reduces oxidative stress	Relieves pain, protects nerves	Lack of clinical evidence, potential adverse effects	[37, 38]
Vitamin drugs	Methylcobalamin	Wide range of indications, provides nutrition and can ameliorate nerve damage by providing antioxidants and neuroprotectants	Safe, cheap, and convenient	Efficacy and safety of some vitamin drugs are still uncertain, poorly effective treatment	[39]
Herbal medicines	Docosahexaenoic acid and α‐lipoic acid dexanabinol cannabinoids paeoniflorin	Reduces inflammation, improves nerve conduction, provides nutritional supplementation, increases antioxidants, and promotes nerve regeneration	Few side effects and long‐lasting efficacy	Active ingredients are complex and uncertain, with issues of confidence and standardization	[31, 40, 41, 42, 43, 44]

Abbreviation: CIPN, chemotherapy‐induced peripheral neuropathy.

### 4.1 Treatment drugs

#### 4.1.1 Duloxetine

Duloxetine is a new selective serotonin and norepinephrine reuptake inhibitor, which has good efficacy and tolerance for treating various types of chronic pain, and is recommended by the International Pain Society for the treatment of neuropathic pain.^31^ The recent clinical practice guidelines of the American Society of Clinical Oncology (ASCO) on the prevention ^45^ and management of CIPN in adult cancer survivors, the European Society of Oncology, and the European Society of Neuro‐Oncology (EANO) clinical practice guidelines on the diagnosis, prevention, treatment, and follow‐up of CIPN provide only moderate recommendations for the use of duloxetine as a symptomatic treatment.^32^ Some studies have found that duloxetine has significant advantages in reducing pain scores and improving function and quality of life in patients with CIPN.^40^

#### 4.1.2 Docosahexaenoic acid and α‐lipoic acid

It has been shown that α‐lipoic acid protects sensory neurons by its antioxidant and mitochondrial regulatory functions and may increase the expression of frataxin (an essential mitochondrial protein with antioxidant and chaperone properties), which may reduce the risk of peripheral neurotoxicity in patients receiving chemotherapy.^46^ A phase II prospective study was performed to evaluate whether the use of a nutritional drug (containing docosahexaenoic acid and α‐lipoic acid) for the first 6 months of bortezomib treatment in 33 patients with a first diagnosis of MM could prevent the development of neurotoxicity. At 6 months, 8 patients had no CIPN, 10 patients had CIPN graded as grade 1, 1 of which was associated with pain, and 17 patients did not report pain symptoms. Although this is a preliminary study with a small number of subjects and a short follow‐up period, and therefore has some limitations, this data seems to indicate that the supplement has the potential to be tested in the future.^41^, ^42^

#### 4.1.3 Methylcobalamin

Methylcobalamin is an endogenous B12 vitamin that promotes axonal transport function and axonal regeneration and has an inhibitory effect on drug‐induced neurodegeneration. A single‐center randomized clinical trial of BIPN in MM patients treated with high‐dose intravenous methylcobalamin (HDIME) found that the incidence of BIPN in the HDIME group was significantly lower than that in the control group; the incidence of grade 2 or grade 3 and above BIPN in the HDIME group was significantly lower compared with the control group. This study showed that HDIME has good efficacy in preventing BIPN and has no serious side effects.^39^

#### 4.1.4 Glutathione

Glutathione is a tripeptide compound containing the sulfhydryl group, and it is the main tripeptide thiol in cells. In one study, in MM patients, 2.4 g of glutathione was administered intravenously once daily 2–3 days before chemotherapy in combination with 500 μg of methylcobalamin every other day until the end of the chemotherapy cycle. Patients who were not treated with this regimen were designated as controls. The results showed that the incidence of CIPN, especially grade 2 and 3 CIPN, was significantly lower in the test group compared to the control group.^37^ Electrophysiological studies have shown that the prototype of glutathione can prevent TIPN, which has been widely used in clinical practice.^38^

#### 4.1.5 Dexanabinol

Model prediction shows that the combined inhibition of TNF α, NMDA receptor, and reactive oxygen species can prevent neuronal apoptosis induced by proteasome inhibitors. Dexanabinol, a synthetic cannabinoid derivative and a three‐target inhibitor, partially restored the decrease of proximal action potential amplitude and distal nerve conduction velocity induced by bortezomib in vitro and prevented mechanical abnormal pain and thermal hyperalgesia induced by bortezomib in rats, including partial recovery of nerve fiber density in the epidermis.^15^ Because it is safe in humans and has been found to be effective in BIPN, dexanabinol may be a treatment option for BIPN.

#### 4.1.6 Gabapentin and pregabalin

In clinical practice, gabapentin and pregabalin are the most commonly used drugs for the management of PN symptoms in patients with MM. MM patients with symptoms of sensory allergy, tingling, or even mild pain benefited from the gabapentin trial. Starting with a low dose to avoid symptomatic drowsiness or dizziness and increasing the planned dose in increments to a maximum dose of 3600 mg per day (if tolerated) is a reasonable approach based on anecdotal experience.^8^ It was found that a single administration of pregabalin (3 mg/kg) and mexiletine (100 mg/kg) was allobiotic against bortezomib‐induced mechanical abnormal pain.^47^ Furthermore, the antihomeopathic effect of gabapentin on bortezomib‐induced mechanical abnormal pain was inhibited by norepinephrine but not by 5‐hydroxytryptamine and spinal cord depletion.^34^ This descending pain inhibition system may also be involved in the analgesic effect of pregabalin on BINP. In a retrospective study, a marked improvement in neurological symptoms during pregabalin treatment was observed, despite the presence of distal sensory axon neuropathy.^35^ Several studies have shown that administration of gabapentin can alleviate symptoms related to CIPN, including neuropathic pain and neurological impairment.^36^ Patients who do not respond well to gabapentin may be significantly improved clinically by switching to pregabalin. Compared to gabapentin, pregabalin has a faster absorption rate, a higher maximum absorption rate, and a higher bioavailability.^48^ Further studies should examine the different efficacy of these drugs in the treatment of CIPN symptoms, especially for pain.

#### 4.1.7 Amitriptyline

In a 12‐month pilot study, a total of 44 patients with BIPN were enrolled and all received topical amitriptyline. At the 1‐year study visit, half of the patients (22/44, 50%) were still using amitriptyline cream, while nine patients (20%) discontinued amitriptyline after 1 month of treatment due to complete pain relief, and the symptoms associated with CIPN did not recur. This prospective pilot study showed that topical amitriptyline at 10% appears to be a first‐line candidate for the treatment of BIPN, and it may allow patients to continue chemotherapy at effective doses. The results deserve to be confirmed in clinical trials.^33^

#### 4.1.8 Cannabinoids

It has been shown that cannabinoids can inhibit neuropathic pain caused by traumatic injury, toxic injury, and metabolic changes, although the mechanism of action of cannabinoid receptors in this process is not fully understood. Indeed, while the relevance of activation of DRG neurons by CB1 receptor (CB1 cannabinoid receptors, CB1R) to explaining the cannabinoid antinociceptive effects has been demonstrated by site‐specific drug administration and tissue‐selective knockdown, the main sites of CB2 receptor (CB2 cannabinoid receptors, CB2R) remain unclear.^43^ Mulpuri et al.^49^ found that local or systemic application of 4‐{2‐[‐(1E)‐1[(4‐propylnaphthalen‐1‐yl)methylidene]‐1H‐inden‐3‐yl]ethyl} morpholine (PrNMI) dose‐dependently suppressed CIPN mechanical and cold allodynia without any CNS side effects. Administration of bortezomib induces increased expression of the atypical cannabinoid receptor TRPV1 and an increase in the number of CB1R‐ and CB2R‐positive DRG neurons in the DRG and spinal cord dorsal horn. So cannabinoids are likely to be effective drugs for the effective treatment of BIPN.^43^

#### 4.1.9 Paeoniflorin

Paeoniflorin is a bioactive compound isolated from the Chinese herbal medicine plant *Paeonia lactiflora*, which has a wide range of anti‐inflammatory, analgesic and immunomodulatory effects.^50^ In addition, paeoniflorin is a potential neuroprotective agent, which has been proved to reduce neuronal damage by reducing the secretion of IL‐6 and other inflammatory factors or reducing the level of autophagy.^51^, ^52^, ^53^ Recent studies have confirmed that paeoniflorin significantly improves BIPN, which may regulate PARKIN‐mediated mitochondrial autophagy and mitochondrial damage by reducing IL‐6 levels.^44^ However, this result has verified only the protective effect of paeoniflorin, which may be due to counteracting neuroinflammation and reducing autophagy injury. Further experiments are needed to confirm the long‐term therapeutic effect of paeoniflorin in BIPN.

Although there are many drugs used clinically for the treatment of MM chemotherapy‐induced peripheral neuropathy, their efficacy is still poor, mainly because the mechanisms are not fully understood. Therefore, it is still necessary to continue to explore the mechanism and further research on the drugs.

### 4.2 Nondrug treatment

#### 4.2.1 Exercise

Exercise may be an effective nonpharmacological treatment for chemotherapy‐related pain, and its positive effect on CIPN still needs further study. However, it is reasonable to consider this therapy due to the relatively small number of exercise‐related side effects.^54^ At present, most of the hypotheses stem from the relationship between exercise and the regulation of inflammatory cytokines, and the level of inflammatory cytokines may be upregulated by chemotherapy drugs. For example, IL‐6 is the most concerned pro‐inflammatory cytokine and is thought to be associated with pain in independent studies.^55^ The proposed preventive mechanism involving IL‐6 is based on the evidence that exercise causes muscles to release IL‐6, which in turn promotes the negative feedback release of anti‐inflammatory mediators, resulting in balance.^54^ Studies suggest that exercise and endogenous cannabinoid release appear to be closely related, and the hypothesis that circulating endogenous cannabinoids coordinate system‐wide responses to energy production, consumption, and storage suggests that increased energy utilization leads to higher circulating levels of endogenous cannabinoids to replenish energy stores and that the increased amount of circulating endogenous cannabinoids after exercise may come from skeletal muscle.^56^

#### 4.2.2 Acupuncture treatment

Acupuncture treatment can significantly reduce CIPN, including neurological symptoms (pain, tingling, and numbness), and improve quality of life and nerve conduction. Therefore, acupuncture is used to treat or prevent CIPN. Acupuncture is safe and feasible and can improve many of the symptoms associated with BIPN or TIPN, especially numbness and tingling in the hands and feet, cold sensitivity, and unpleasant sensations.^57^ Acupuncture may help with nerve repair by increasing blood flow to the extremities. A 6‐week course of acupuncture significantly improves symptoms of pain, numbness, and tingling in patients with Class II CIPN.^58^ The results of a randomized controlled trial showed that patients treated with mecobalamin alone and patients treated with acupuncture in combination with mecobalamin had significant relief of pain and numbness, but acupuncture combined with mecobalamin had a more significant reduction in pain scores and a significant improvement in satisfaction with activities of daily living than mecobalamin alone.^7^ A recent study has shown that auricular acupuncture is an effective, relatively safe, and inexpensive treatment for CIPN, and that it can be used early in the administration of chemotherapy and in a variety of other conditions.^59^

#### 4.2.3 Nerve block

Nerve block refers to the injection of anesthetics or physical stimulation around the nerve trunk, plexus, or ganglion to block nerve conduction, which has the advantages of accurate positioning and relatively few adverse effects. A variety of treatments, including paravertebral nerve blocks, selective nerve root blocks, and sympathetic nerve blocks, are now widely used in the treatment of peripheral neuralgia. Peripheral nerve blocks with local anesthetics are effective in relieving peripheral nerve pain.^60^ Therefore, its effectiveness in MM chemotherapy‐induced peripheral neuropathy deserves further investigation.

#### 4.2.4 Electrical stimulation

It has been reported that electrical stimulation therapy can promote axonal regeneration of nerve cells and functional recovery after peripheral nerve injury and also reduce neuropathic pain. The therapeutic mechanism of electrical stimulation can be summarized as follows: electrical stimulation can reduce neuropathic pain and improve nerve function by inhibiting synaptic stripping and DRG hyperexcitability. Electrical stimulation at peripheral nerve injury sites can accelerate nerve regeneration, while electrical stimulation at target organ sites can reduce the atrophy of denervated skeletal muscle and promote the recovery of sensory function.^61^ Scrambler therapy uses scrambler devices to transmit electrical stimulation to the skin. Nonrandomized pilot studies have shown that this therapy is beneficial in reducing pain scores, numbness, and tingling in CIPN.^62^ Further research should be conducted to determine whether scrambler therapy is a viable treatment option for those with CIPN symptoms, especially peripheral neuropathy due to chemotherapy for MM.

All of the above nonpharmacological therapies are worth exploring, which not only requires enhanced multidisciplinary cooperation but also requires doing early interventions and interventions of these therapies, which may have certain expected effects but is still a path that needs to be explored in the long term.

## 5 CONCLUSION AND PROSPECTS

Although drugs such as duloxetine, docosahexaenoic acid and α‐lipoic acid, methylcobalamin, glutathione, dexanabinol, anticonvulsants, antidepressants, cannabinoids, and paeoniflorin, as well as nonpharmacological treatments, have some efficacy in CIPN, there are no highly recommended methods to prevent or treat the development of CIPN, and detailed studies on the biological mechanisms of CIPN are needed to find the direction of treatment. Despite a growing body of literature on the pathophysiological mechanisms and treatment of CIPN, new treatment options remain limited and ineffective, with some patients experiencing difficulty controlling their symptoms and severely compromising their quality of life. Guided by the prior research literature, new approaches that may help treat CIPN will slowly emerge. With the deepening of the research on the occurrence mechanism and prevention measures of CIPN, the efficacy of a series of drug and nondrug therapies has gradually been defined, but to achieve a better, more comprehensive, and more satisfactory efficacy, we still need to conduct a deeper and more extensive exploration, with a long way to go. The existing treatment modalities are mainly to improve the clinical symptoms of the patients. At the same time, since both pharmacological and nonpharmacological treatments have a certain degree of efficacy, it remains to be verified whether a reasonable combination of these treatment modalities will help the patients to obtain a better therapeutic effect.

## AUTHOR CONTRIBUTIONS

Dan Wen and Yonghuai Feng contributed to the main ideas of this review, leading to the submission of the paper and the collection of resources. Song Cao contributed to the organizational thinking and revision of the paper, finalized the manuscript, and approved the final version for review.

## CONFLICT OF INTEREST STATEMENT

Song Cao is the editorial member of Ibrain, who has not been involved in the peer review process.

## ETHICS STATEMENT

Not applicable.

## Data Availability

The data reported in this study are available from the Lead Contact on request.
